# Supplementary material for: EnzML: multi-label prediction of enzyme classes using InterPro signatures
Source: BMC Bioinformatics. 2012 Apr 25;13:61. doi: 10.1186/1471-2105-13-61 (PMC3483700; doi:10.1186/1471-2105-13-61)
Supplement: Addtional file 5 — The Java code to format the data files, evaluate and predict. The file enzml_java_code.tar.gz contains the Java code used to format database data to ARFF and XML formats, to execute cross and train-test (jackknife) evaluations and to record evaluation results to database. More information is included in the readme.txt file and the Javadoc files. The code can be used with a MySQL database. To use a different database software, other JDBC drivers might be required. [file 1471-2105-13-61-S5.gz › java_code/utils/doc/uk/ac/ed/inf/utils/LogUtils.html]

LogUtils


---


|  |  |  |
| --- | --- | --- |
| **Overview** \| **Package** \| **Class** \| **Use** \| **Tree** \| **Deprecated** \| **Index** \| **Help** | |  |
| **PREV CLASS**   **NEXT CLASS** | **FRAMES**    **NO FRAMES**     **All Classes** |  |
| SUMMARY: NESTED \| FIELD \| CONSTR \| METHOD | DETAIL: FIELD \| CONSTR \| METHOD |  |


---


## uk.ac.ed.inf.utils Class LogUtils

```
java.lang.Object
  uk.ac.ed.inf.utils.LogUtils
```

---

``` public class LogUtils extends java.lang.Object ```

Utilities to log error messages

**Version:**
:   27 Feb 2009

**Author:**
:   Luna De Ferrari luna.deferrari-at-ed.ac.uk

---

**Constructor Summary**

`LogUtils()`


| **Method Summary** | |
| --- | --- |
| `static java.lang.String` | `error(java.lang.Object o)` |
| `static void` | `error(java.lang.Object o, java.lang.String text)` |
| `static java.lang.String` | `error(java.lang.String s)` |
| `static java.lang.String` | `log(java.lang.Object o)` |
| `static void` | `log(java.lang.Object o, java.lang.String text)` |
| `static void` | `logAndPrint(java.io.File logFile, java.lang.String text)`             Logs some text to file AND prints it to screen. |
| `static void` | `redirectStdErr(java.io.File file)`             Redirect stderr to file |

**Methods inherited from class java.lang.Object**

`equals, getClass, hashCode, notify, notifyAll, toString, wait, wait, wait`

**Constructor Detail**

### LogUtils

```
public LogUtils()
```


**Method Detail**

### error

```
public static java.lang.String error(java.lang.Object o)
```

---


### error

```
public static void error(java.lang.Object o,
                         java.lang.String text)
```

---


### error

```
public static java.lang.String error(java.lang.String s)
```

---


### log

```
public static java.lang.String log(java.lang.Object o)
```

---


### log

```
public static void log(java.lang.Object o,
                       java.lang.String text)
```

---


### logAndPrint

```
public static void logAndPrint(java.io.File logFile,
                               java.lang.String text)
```

:   Logs some text to file AND prints it to screen. If the file is null, it
    prints to screen only.

    :   **Parameters:**: `logFile` - the log file (can be null): `text` - the message to be logged

---


### redirectStdErr

```
public static void redirectStdErr(java.io.File file)
```

:   Redirect stderr to file


---


|  |  |  |
| --- | --- | --- |
| **Overview** \| **Package** \| **Class** \| **Use** \| **Tree** \| **Deprecated** \| **Index** \| **Help** | |  |
| **PREV CLASS**   **NEXT CLASS** | **FRAMES**    **NO FRAMES**     **All Classes** |  |
| SUMMARY: NESTED \| FIELD \| CONSTR \| METHOD | DETAIL: FIELD \| CONSTR \| METHOD |  |


---
